# Cold atmospheric plasma affects metabolic processes in malignant melanoma

**DOI:** 10.1186/s12935-026-04448-3

**Published:** 2026-08-22

**Authors:** Erika Rein, Tom Zimmermann, Stefan Fischer, Zubeir El Ahmad, R. Verena Taudte, Arne Gessner, Martin F. Fromm, Melanie Kappelmann-Fenzl, Anja Bosserhoff

**Affiliations:** 1 Institute of Biochemistry, University of Erlangen-Nürnberg, 91054 Erlangen, Germany; 2 Faculty of Computer Science, Deggendorf Institute of Technology, 94469 Deggendorf, Germany; 3 Institute of Experimental and Clinical Pharmacology and Toxicology, University of Erlangen-Nürnberg, 91054 Erlangen, Germany; 4 FAU NeW - Research Center New Bioactive Compounds, University of Erlangen-Nürnberg, 91054 Erlangen, Germany; 5 Comprehensive Cancer Center Erlangen-EMN (CCC ER- EMN), 91054 Erlangen, Germany; 6 Comprehensive Cancer Center Alliance WERA (CCC WERA), 91054 Erlangen, Germany; 7 Bavarian Cancer Research Center (BZKF), 91054 Erlangen, Germany

**Keywords:** Cold atmospheric plasma, Malignant melanoma, Metabolism, Protein nitration

## Abstract

**Background:**

Partially ionized gas, also known as cold atmospheric plasma (CAP), is reported to have anti-tumor specific properties via reactive oxygen and nitrogen species (RONS) in different tumor entities. Many CAP effects have already been studied, but the exact influence on metabolism has yet to be defined.

**Methods:**

Analyses of metabolic substrates, metabolic pathways, genes, mRNA and nitration were performed to investigate CAP-treated and untreated melanoma and non-malignant cells.

**Results:**

We discovered a dose-dependent downregulation of oxidative phosphorylation and glycolysis in CAP-treated compared to untreated melanoma cells, whereas for non-malignant cells we saw no change in the oxidative phosphorylation and even an increase in the glycolysis. Gene set enrichment analyses demonstrated a downregulation of gene sets associated with metabolic processes in melanoma cells. These effects could be explained by posttranslational modification via tyrosine nitration, induced by RONS. A higher induction of protein nitration was found in CAP-treated melanoma cells opposed to non-malignant cells. This difference may contribute to the antitumor-specific effects of CAP.

**Conclusion:**

This study shows different molecular and metabolic responses of melanoma and non-malignant cells in vitro. It thereby contributes to a better understanding of the antitumor-specific effects of CAP and facilitates the development of CAP-based treatments.

**Supplementary Information:**

The online version contains supplementary material available at 10.1186/s12935-026-04448-3.

## Background

The term “plasma” was introduced by the chemist Irving Langmuir in 1928 [1] and is described as the fourth state of matter. Depending on the degree of ionization three different types are differentiated: High temperature plasma (< 10^8^ K) is fully ionized, thermal plasma is almost fully ionized [2] and lastly cold atmospheric plasma (CAP) is weakly ionized plasma at atmospheric pressure and room temperature [3]. Thus, CAP can be utilized to treat biological samples without thermal stress.

The CAP-produced reactive oxygen and nitrogen species (RONS) [4] subsequently reach intracellular targets for example by diffusing across the plasma membrane or by permeating through membrane channels such as aquaporins [5]. RONS lead to various implications on tumor associated processes such as apoptosis and cell proliferation [6, 7]. It is known that RONS can cause oxidative stress in cells [7], but are also important for physiological functions (e.g. healing) and signaling [8]. Furthermore, CAP also leads to the presence of an electric field and UV radiation [9].

Today, CAP is applied in the field of dermatology to support of wound healing processes [6] or for disinfection [10] and was investigated in the research field, called “plasma oncology”. In this regard CAP was shown to have antitumor-specific properties in at least 20 different cancer entities, including melanoma, leukemia and cervical cancer [11]. Most of the studies were performed in vitro and demonstrated different molecular effects in cancer cells after CAP treatment. Cancer cells responded with different types of cell death, DNA damage, mitochondrial damage [12], senescence [13] and a higher sensitivity to drugs [14]. Also, in vivo studies in mice displayed an anti-cancer effect of CAP, as the tumor size decreased while the survival time increased of malignant glioma and neuroblastoma xenografts in immunodeficient mice [15, 16]. Furthermore, a few successful clinical tests of CAP were performed in the past few years, like a stage IV colon cancer patient treated with CAP during surgery showed no more detectable tumor after three months [17]. Additionally, Metelmann et al. used CAP to treat 12 patients with squamous head and neck cell carcinoma. Overall, the patients felt less pain, the superficial tumor decreased partially and wound healing process improved [18]. Since CAP negatively influences tumors in vivo, it is a promising treatment option, for example after removal of tumors during surgery, for superficial tumors like melanoma or as an additional treatment for combinational therapy with other anti-cancer agents [19].

In contrast to malignant cells, non-malignant cells are not negatively affected by CAP. It even has been shown to have positive effects on non-malignant cells. CAP supported cell migration and proliferation [6] and elevated the gene expression of inflammatory cytokines and the wound healing without cell death induction in keratinocytes [20]. CAP also promoted angiogenesis through its effects on keratinocytes, fibroblasts and endothelial cells [21]. One predisposition for the antitumor-specific effect could be the differential metabolic properties between cancer and non-malignant cells, which could affect their response to treatments such as CAP. While normal cells typically maintain homeostatic control over their metabolism, cancer cells often show increased glycolysis and produce more lactic acid, even under aerobic conditions, a phenomenon known as the “Warburg effect” [22]. Therefore, it is important to consider the metabolic differences between these cell types to understand the full potential of CAP treatment against cancer.

As briefly mentioned, several aspects of CAP treatment have been already investigated, but studies directly addressing metabolic alterations remain comparatively limited. Nevertheless, some metabolomic analyses have demonstrated that CAP treatment induces changes in cellular metabolism in cancer. For example, in glycolysis [23] and amino acid metabolism [24]. Although CAP-induced metabolic alterations have been reported, the molecular mechanisms linking CAP-derived RONS to metabolic reprogramming remain incompletely understood. Furthermore, cell-type specific metabolic responses are largely unknown.

Therefore, we aimed to investigate the effect of CAP on cellular metabolic processes in malignant melanoma in contrast to melanocytes and fibroblasts. We assumed that the metabolism of melanoma cells was impaired by CAP as opposed to non-malignant cells.

## Materials and methods

### Cell culture

The melanoma cell line Mel Juso was cultivated in RPMI 1640 Medium (with 2% sodium bicarbonate), the cell line Mel Im in DMEM low-glucose medium and the fibroblasts Bj1 in DMEM high-glucose medium. Following supplements were added to these cell culture media: 1% penicillin/streptomycin and 10% FCS. The normal human epidermal melanocytes (NHEM) were purchased from Lonza (Basel, Switzerland) and required MBM^TM^−4 medium with MGM^TM^−4 SingleQuots™ supplements. All cells were incubated at 37 °C. The Mel Juso and Bj1 cell lines in 5% CO_2_, while the Mel Im and NHEM were cultivated at 8% CO_2_. When reaching about 80% confluence, the cells were passaged. For that the cells were detached using 0.05% trypsin/0.02% EDTA in PBS and were resuspended after centrifugation in their respective medium for further cultivation or were counted using a Neubauer counting chamber for experiments. All cell lines were regularly tested for mycoplasma contamination. All media (besides for the NHEM) were acquired from Sigma Aldrich (Burlington, USA).

**Fig. 1:**
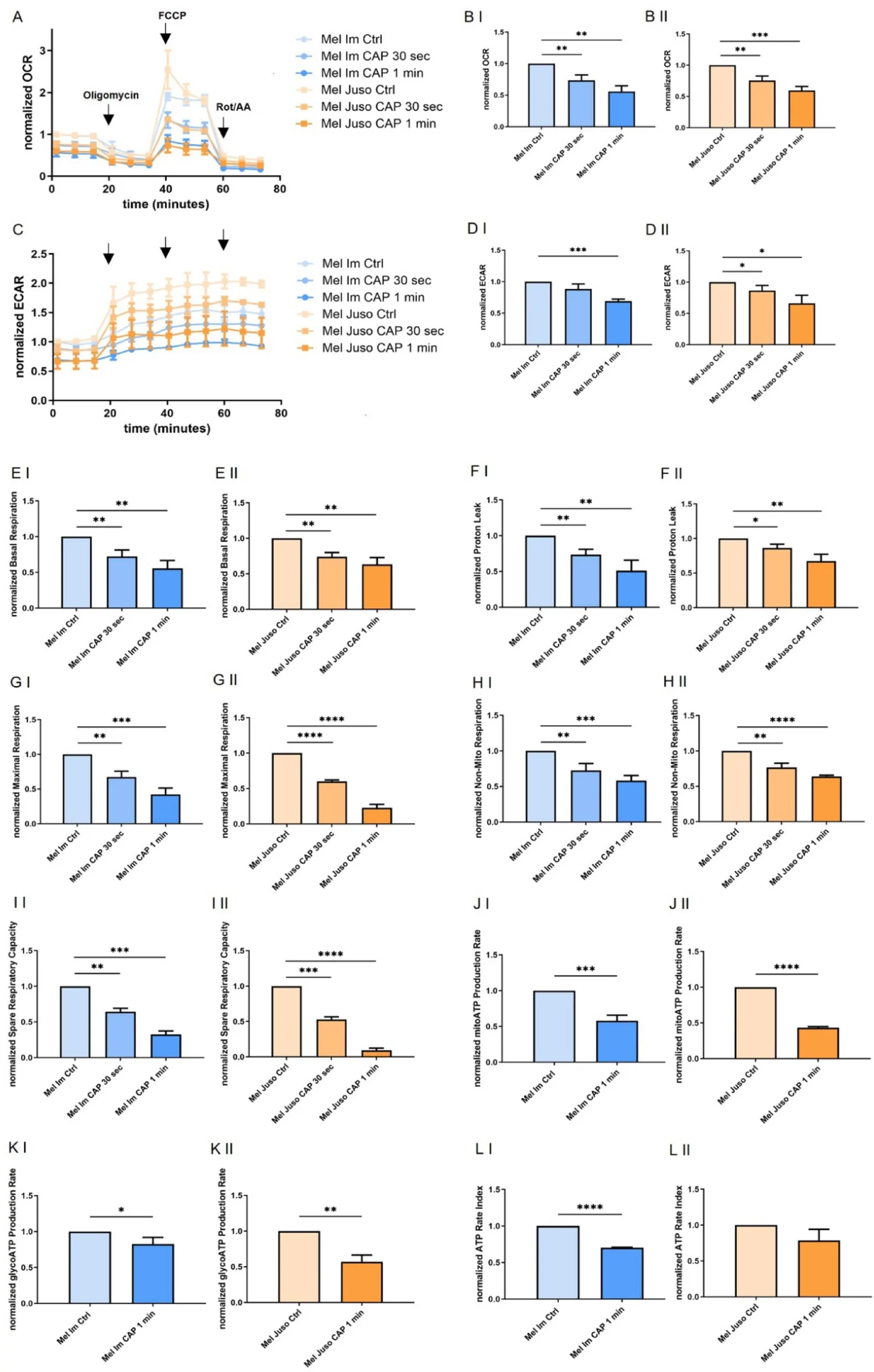
Real-time metabolic measurement using the Seahorse XF HS Mini Analyzer 24 h after 30 s or 1 min CAP treatment (**A-I**: Mito Stress test, **J-L**: ATP Rate Assay). (**A**) Kinetic curve demonstrating the oxygen consumption rate (OCR) against the time in minutes in Mel Im and Mel Juso Ctrl and CAP. Arrows indicate the inhibitor injections: Carbonylcyanide-4-(trifluoromethoxy)phenylhydrazone (FCCP) and Rotenone/Antimycin A (Rot/AA) (**B I-II**) Bar chart depicting the normalized downregulated oxygen consumption rate (OCR) in CAP-treated Mel Im and Mel Juso compared to Ctrl. (**C**) Kinetic curve showing the extracellular acidification rate (ECAR) against the time in minutes in Mel Im and Mel Juso Ctrl and CAP. Arrows indicate the inhibitor injections (see 1 A). (**D I-II**) Bar chart of the normalized downregulated extracellular acidification rate (ECAR) in CAP-treated Mel Im and Mel Juso compared to Ctrl. (**E I-II**) Bar charts of the normalized decreased basal respiration, (**F I-II**) proton leak, (**G I-II**) maximal respiration, (**H I-II**) non-mito respiration and (**I I-II**) spare respiratory capacity in CAP-treated Mel Im and Mel Juso compared to Ctrl. (**J I-II**) Bar charts of the normalized decreased mitoATP Production Rate and (**K I-II**) glycoATP Production Rate in CAP-treated Mel Im and Mel Juso. (**L I-II**) Bar charts of the ATP Rate Index in Mel Im and Mel Juso control and CAP. blue tones = Mel Im; orange tones = Mel Juso; CAP treatments were normalized to the respective control and statistically analyzed using an unpaired t-test; * *p* < 0.05; ** *p* < 0.005; *** *p* < 0.0005; **** *p* < 0.0001; *n* = 3; mean ± standard deviation

### CAP treatment

For the CAP treatment the plasma care^®^ device from Terraplasma Medical GmbH (Garching, Germany) was applied. This device belongs to the surface micro discharge technology. It has an electrode, which leads to micro discharges of the surrounding air under high voltage (3,5 kV). Prior to the treatment, the device was warmed up for 5 min without a sample to guarantee an optimal working temperature for a reliable treatment. A custom, 3D-printed spacer accomplished a tight fit of a single 35 mm cell culture dish to the device. Thus, the distance between the cells and the plasma device was constant (approximately 10 mm) and comparable to previous studies [19, 25]. 200,000 cells (80% confluency) were seeded in 35 mm cell culture dishes prior, washed with PBS and treated with a reduced amount of medium with CAP for 30 s or one minute, since CAP is limited by its penetration depth [19]. Afterwards, 1 ml of the respective complete culture medium was added and the cells were incubated for different time points at 37 °C. The control cells were handled the same way, but instead of CAP, they were incubated without medium for 30 s or one minute. Afterwards, a one or 24 h incubation followed before the cells were used for further experiments. The experimental setup is illustrated in supplement 1a. Furthermore, since cell viability is affected by the CAP treatment, cell counts after one minute CAP treatment have been conducted and depicted in supplement 1b for every cell line.

**Fig. 2:**
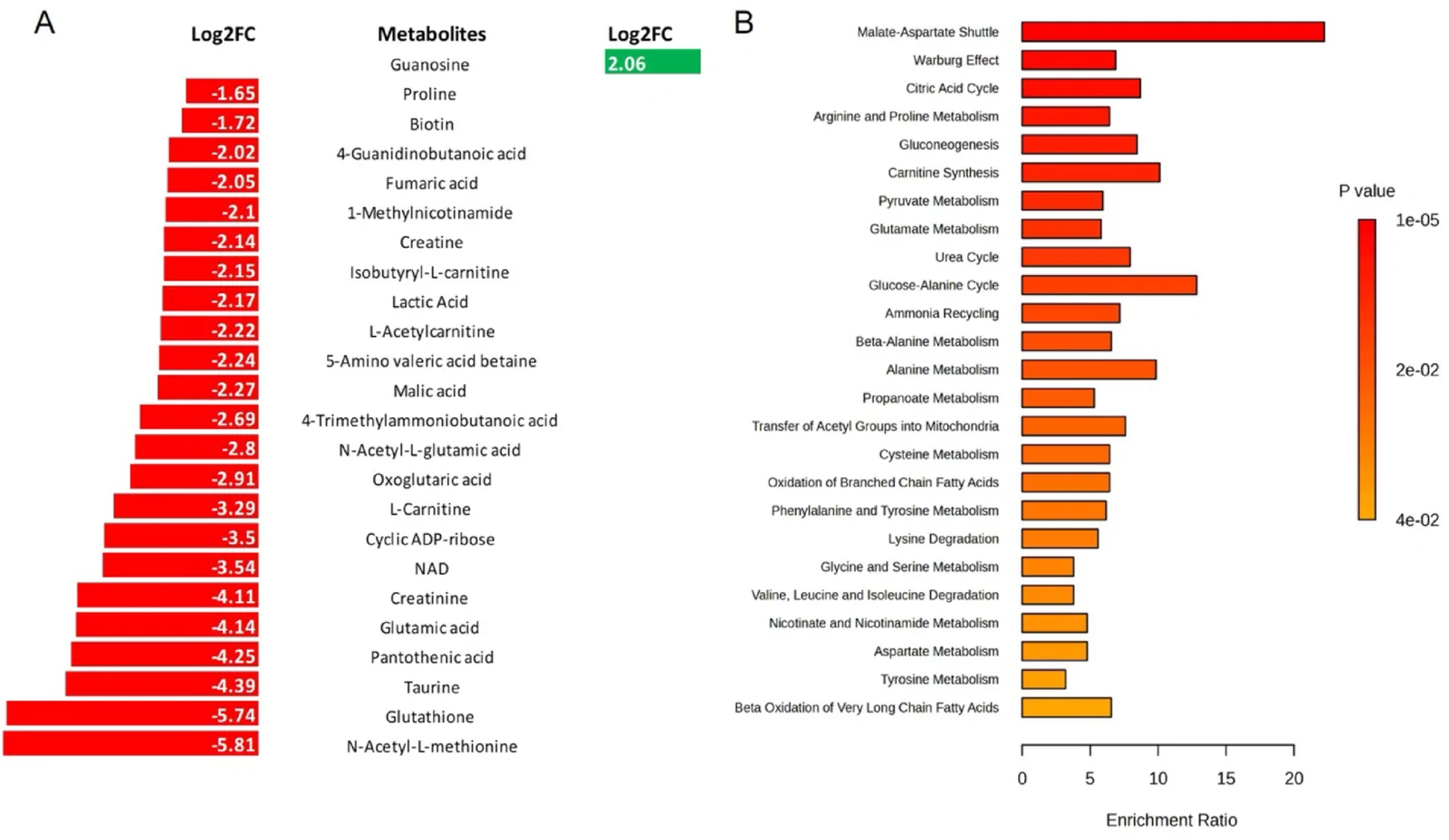
Differential metabolite concentration one hour after CAP treatment in Mel Juso cells. (**A**) Significantly differential quantity metabolites in CAP-treated Mel Juso detected by LC/MS (red = decrease, green = increase. (**B**) Significantly dysregulated pathways based on the LC/MS analysis of metabolites of CAP-treated Mel Juso after 1 h post-treatment incubation using MetaboAnalyst 6.0 enrichment analysis

### Analysis of mRNA expression using real-time PCR

Total RNA isolation was performed using E.Z.N.A.^®^ Total RNA Kit II from Omega Bio-Tek (Norcross, USA) using the manufacturer’s instructions, followed by reverse transcription into cDNA (as described elsewhere [26]). Real-time PCR was then performed using LightCycler^®^ 480 II devices from Roche (Basel, Switzerland) using an annealing temperature of 60 °C. Forward and reverse primers were ordered from Sigma Aldrich (Burlington, USA) (sequences are shown in supplement 2). Beta-actin was used as a reference gene for normalization.

### Measurement of protein nitration via western blot analysis

Cell pellets were lysed using radio-immunoprecipitation assay buffer from Roche (Basel, Switzerland). Afterwards, the protein concentration was measured by Pierce™ BCA protein assay from Thermo Fisher Scientific (Waltham, USA). Approximately 40 µg protein lysate was loaded per lane for electrophoresis (10% SDS polyacrylamide gel). The protein on the SDS gel was transferred onto PVDF membranes from Bio-Rad (Hercules, USA). The total protein load was visualized by Ponceau S staining. Then, the membranes were blocked using 5% non-fat dried milk (NFDM) in TBST for one hour. Primary antibodies against nitrotyrosine diluted 1:1000 in TBST (06–284), from Sigma Aldrich (Burlington, USA) and GAPDH (glycerinaldehyde-3-phosphate dehydrogenase), diluted 1:2000 in 5% NFDM (2118 S), from Cell Signaling, (Danvers, USA) were incubated on the membrane overnight at 4 °C. After three washing steps with TBST, the secondary anti-rabbit horseradish peroxidase (HRP)-coupled secondary antibodies (1:3000 in TBST) from Cell Signaling (Danvers, USA) were added for one hour at room temperature. The protein lanes were detected using the Clarity™ Western ECL Substrate from Bio-Rad (Hercules, USA) and Chemostar chemiluminescence imager from Intas (Goettingen, Germany). The LabImage software (version 4.2.3) from Kapelan Bio-Imaging GmbH (Leipzig, Germany) was utilized for quantitative analysis of the protein chemiluminescence intensity. Original blots see supplement 1c.

### Glucose measurements

The glucose concentrations in the supernatants of CAP-treated cells were measured utilizing the FreeStyle Precision glucose measurement device from Abbott (North Chicago, USA). Afterwards, the cells were detached and counted using a Neubauer counting chamber for normalization.

**Fig. 3:**
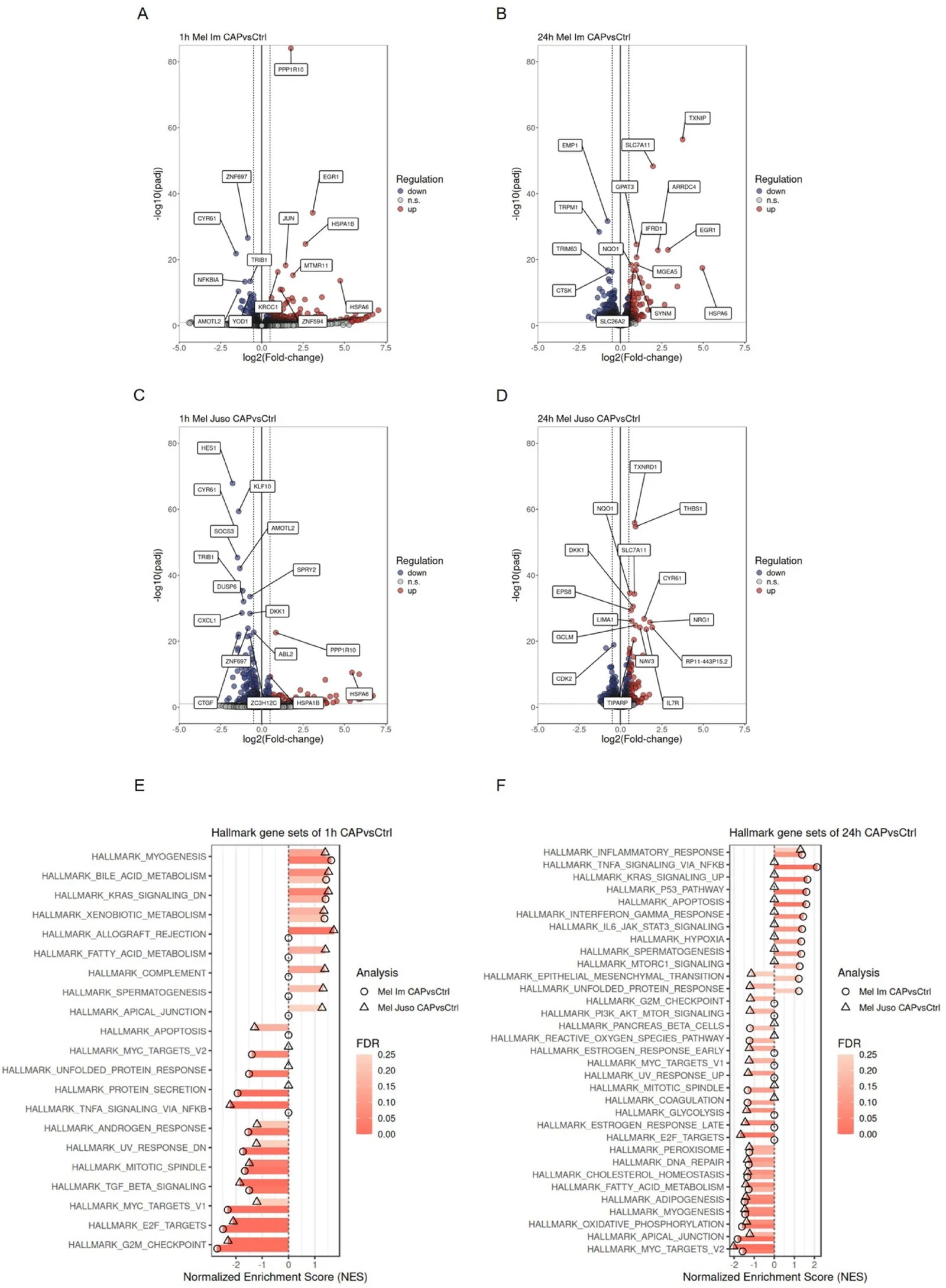
Volcano plots of up- and downregulated genes in CAP-treated (**A**) Mel Im 1 h incubation, (**B**) Mel Im 24 h incubation, (**C**) Mel Juso 1 h incubation and (**D**) Mel Juso 24 h incubation after CAP treatment. Gene Set Enrichment Analysis (GSEA) of CAP-treated Mel Im and Mel Juso (**E**) 1 h and (**F**) 24 h after CAP treatment using the hallmark gene set. FDR = false discovery rate; statistic thresholds: adjusted p-value < 0.1; FDR < 0.25

### Glutamate/Glutamine measurements

For the analysis of glutamate and glutamine concentration in the supernatant of CAP-treated cells the Promega Glutamine/Glutamate-Glo™ Kit from Promega, (Madison, USA) was used and executed as the providers manual states. The cell number was utilized for normalization.

### Real-time metabolic measurements

Real-time metabolic measurements of the oxygen consumption rate (OCR) and extracellular acidification rate (ECAR) were performed using the Agilent Seahorse XF HS Mini Analyzer from Agilent (Santa Clara, USA). The Mito Stress test and ATP Production Rate assay were executed as documented in the manufacturer’s instruction 24 h after CAP treatment. Following inhibitors were used in the mentioned assays: Oligomycin for the inhibition of the ATP synthase, Carbonylcyanide-4-(trifluoromethoxy)phenylhydrazone (FCCP) as an uncoupling agent and Rotenone/Antimycin A (Rot/AA) for the inhibition of complex I and III of the mitochondrial electron transport chain. In the Mito Stress test Oligomycin was used first followed by FCCP and Rot/AA, while for the ATP Production Rate assay, Oligomycin and then Rot/AA were utilized.

### Liquid chromatography-mass spectrometry for untargeted metabolomics analysis

This procedure was performed as previously described [19]. MetaboAnalyst (Version 6.0) was used for enrichment analysis of differential regulated metabolites (*p* < 0.05 and log2fold change > 1.5 and < −1.5). A table of the analyzed metabolites is provided in supplement 3.

**Fig. 4:**
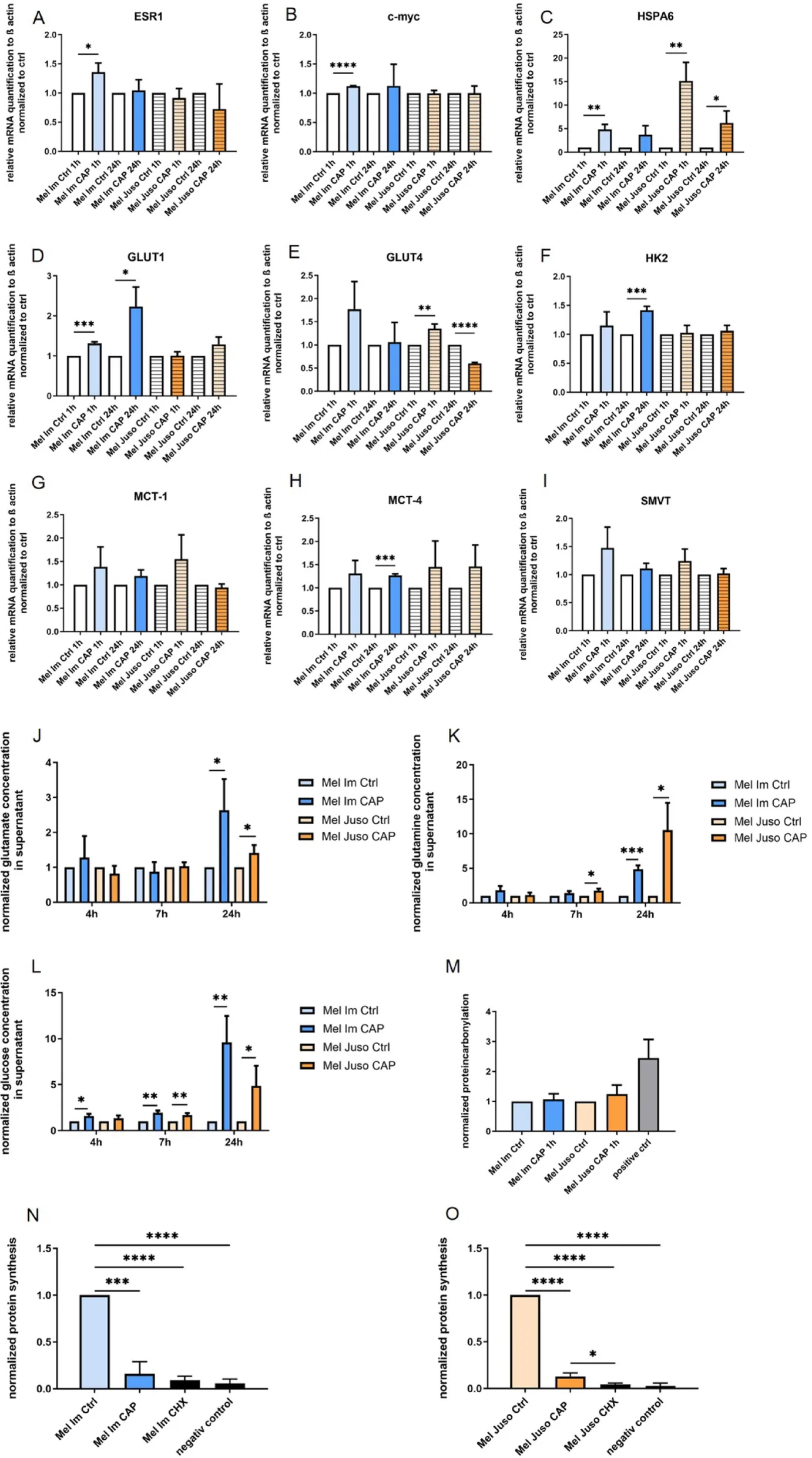
mRNA expression level of (**A**) ESR1 (**B**) c-myc (**C**) HSPA6 (**D**) GLUT1 (**E**) GLUT4 (**F**) HK2 (**G**) MCT-1 (**H**) MCT-4 (**I**) SMVT 1 h and 24 h after CAP treatment in Mel Im and Mel Juso analyzed via qPCR and normalized to the expression of ß-actin. Measurements of the supernatant concentration of (**J**) glutamate (**K**) glutamine and (**L**) glucose after different time points of CAP and Ctrl-treated Mel Im and Mel Juso. (**M**) Analysis of the protein carbonylation content after CAP treatment in melanoma (positive control: H_2_O_2_-treated Mel Im). Normalized decreased protein synthesis 1 h after CAP treatment (**N**) in Mel Im and (**O**) in Mel Juso analyzed via Click-IT™ HPG Alexa Fluor™ 594 Protein Synthesis Assay Kit. CHX=cycloheximide as positive control for protein synthesis inhibition. blue tones: Mel Im; orange tones: Mel Juso; CAP treatment was normalized to the respective control and statistically analyzed using an unpaired t-test * *p* < 0.05; ** *p* < 0.005; *** *p* < 0.0005; **** *p* < 0.0001; *n* = 3; mean ± standard deviation

### RNA sequencing

Total RNA was extracted from treated and untreated Mel Im and Mel Juso cells one hour and 24 h after one minute CAP treatment using the Total RNA kit I from Omega Bio-Tek (Norcross, USA) according to the manufacturer’s instructions. All the RNA samples were examined for integrity and purity with a TapeStation 4200 from Agilent (Santa Clara, USA). Library preparation was performed with three biological replicates using the TruSeq^®^ Stranded Total RNA Library Prep Human/Mouse/Rat Kit from Illumina Inc. (San Diego, USA) according to the manufacturer’s instructions. The resulting libraries were checked for size (200–500 bp) utilizing the TapeStation 4200 Agilent (Santa Clara, USA) and for concentration using a Qubit 4 Fluorometer from Thermo Fisher (Waltham, USA). Sequencing was performed according to the paired-end RNA sequencing protocols from Illumina on a NovaSeq 6000 with a paired-end module from Illumina, Inc. (San Diego, USA). The samples were sequenced from each side of a fragment approximately 100 bp long, with an average number of 20 million reads per sample. Further data pre-processing steps like quality check and mapping of the reads were conducted as described previously [27]. The raw reads were then filtered, normalized and visualized by using R (v4.4.2) [28]. Logarithmic-transformation and differential expression analysis of the sequencing data were performed individually for each cell line according to a standard DESeq2 approach (v.1.44.0) [29] with adjusted p-values calculated using the Benjamini-Hochberg procedure [30]. Differentially expressed genes were defined with an adjusted p-value < 0.1 (padj). Overlaps of significantly regulated genes from Mel Im and Mel Juso libraries were produced, the corresponding log2FoldChange (log2FC) was scaled individually and the top percent of regulated genes were identified by the sum of the corresponding log2FC of Mel Im and Mel Juso cells. Functional bioinformatic data analysis was performed via Gene Set Enrichment Analysis (GSEA) along with the Molecular Signature Database (MSigDB) using classical weighting, log2FC metric, and 1000 permutations of gene sets [31, 32]. GSEA results were considered significant with an FDR < 0.25. Data processing and plots of bioinformatic data were produced by using R packages tidyr (v. 1.3.1) [33] and ggplot2 (v. 4.0.1) [34].

**Fig. 5:**
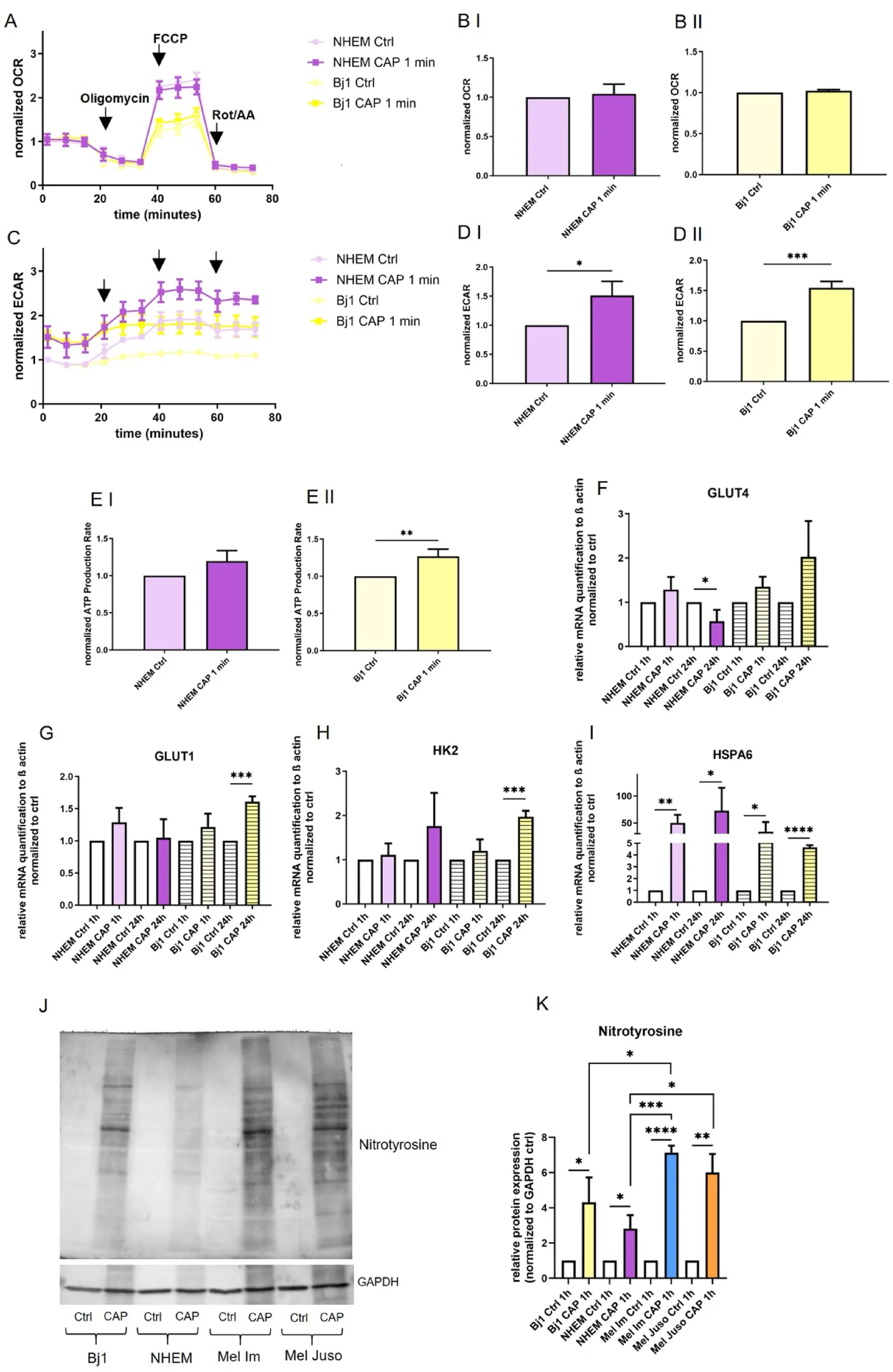
Real-time metabolic measurement using the Seahorse XF HS Mini Analyzer (Mito Stress Test) 24 h after CAP treatment in NHEM and Bj1 cells. (**A**) Kinetic curve demonstrating the oxygen consumption rate (OCR) against the time in minutes in NHEM and Bj1 Ctrl and CAP. Arrows indicate the inhibitor injections. Carbonylcyanide-4-(trifluoromethoxy)phenylhydrazone (FCCP) and Rotenone/Antimycin A (Rot/AA) (**B I-II**) Bar chart depicting the normalized oxygen consumption rate (OCR) in NHEM and Bj1. (**C**) Kinetic curve showing the extracellular acidification rate (ECAR) against the time in minutes in NHEM and Bj1 Ctrl and CAP. Arrows indicate the inhibitor injections (see above). (**D I-II**) Bar chart of the normalized increased extracellular acidification rate (ECAR) in CAP-treated NHEM and Bj1 compared to Ctrl. (**E I-II**) Bar charts of the elevated ATP Production Rate in Bj1 and NHEM (ATP Rate Assay). Normalized mRNA expression of (**F**) GLUT4 (**G**) GLUT1 (**H**) HK2 (**I**) HSPA6 of CAP and Ctrl-treated NHEM and Bj1 cells after 1 h and 24 h incubation via qPCR analysis. Western Blot analysis of (**J**) nitrotyrosine normalized to GAPDH in Bj1, NHEM, Mel Im and Mel Juso. (**K**) Quantification of nitrotyrosine protein expression. blue tone: Mel Im; orange tone: Mel Juso; purple tones: NHEM and yellow tones: Bj1; CAP treatment was normalized to the respective control and statistically analyzed using an unpaired t-test; additionally, the CAP treatments in bar chart K were also compared between two cell lines via an unpaired t-test; * *p* < 0.05; ** *p* < 0.005; *** *p* < 0.0005; *n* = 3; mean ± standard deviation

Although RNA-seq and qPCR are complementary approaches for gene expression analysis, complete concordance between both methods is not always expected. Differences may arise from distinct normalization procedures, sequencing depth, transcript abundance, RNA folding, primer specificity, and the detection of alternative transcript isoforms [35]. Therefore, minor discrepancies in fold changes or statistical significance between both methods are not uncommon.

The normalized counts can be accessed in supplement 4 while the generated heatmaps can be retrieved in supplement 1d-e. Furthermore, a table consisting of the differential expressed genes (with log2FC) can be found in supplement 5.

### Protein carbonylation

The protein carbonylation amount was measured using the Protein Carbonyl Content Assay Kit (ab126287) from Abcam (Cambridge, United Kingdom) in accordance with the manufacturer`s protocol.

### Protein synthesis assay via fluorescence microscopy

The Click-iT^®^ HPG Alexa Fluor^®^ Protein Synthesis Assay Kit (C10429) from Thermo Fisher (Waltham, USA) was used to analyze newly synthesized proteins of one hour CAP-treated and untreated cells in accordance with the manufacturer`s protocol. The fluorescence images were taken via the Leica DMi8 microscope from Leica Microsystems (Wetzlar, Germany) and for the fluorescence data analyses ImageJ (version 1.54p) was utilized. A coverslip without the L-Homopropargylglycine (HPG) was the negative control. Furthermore, cells treated with 10 µM cycloheximide were used as a positive control for the inhibition of protein translation.

**Fig. 6:**
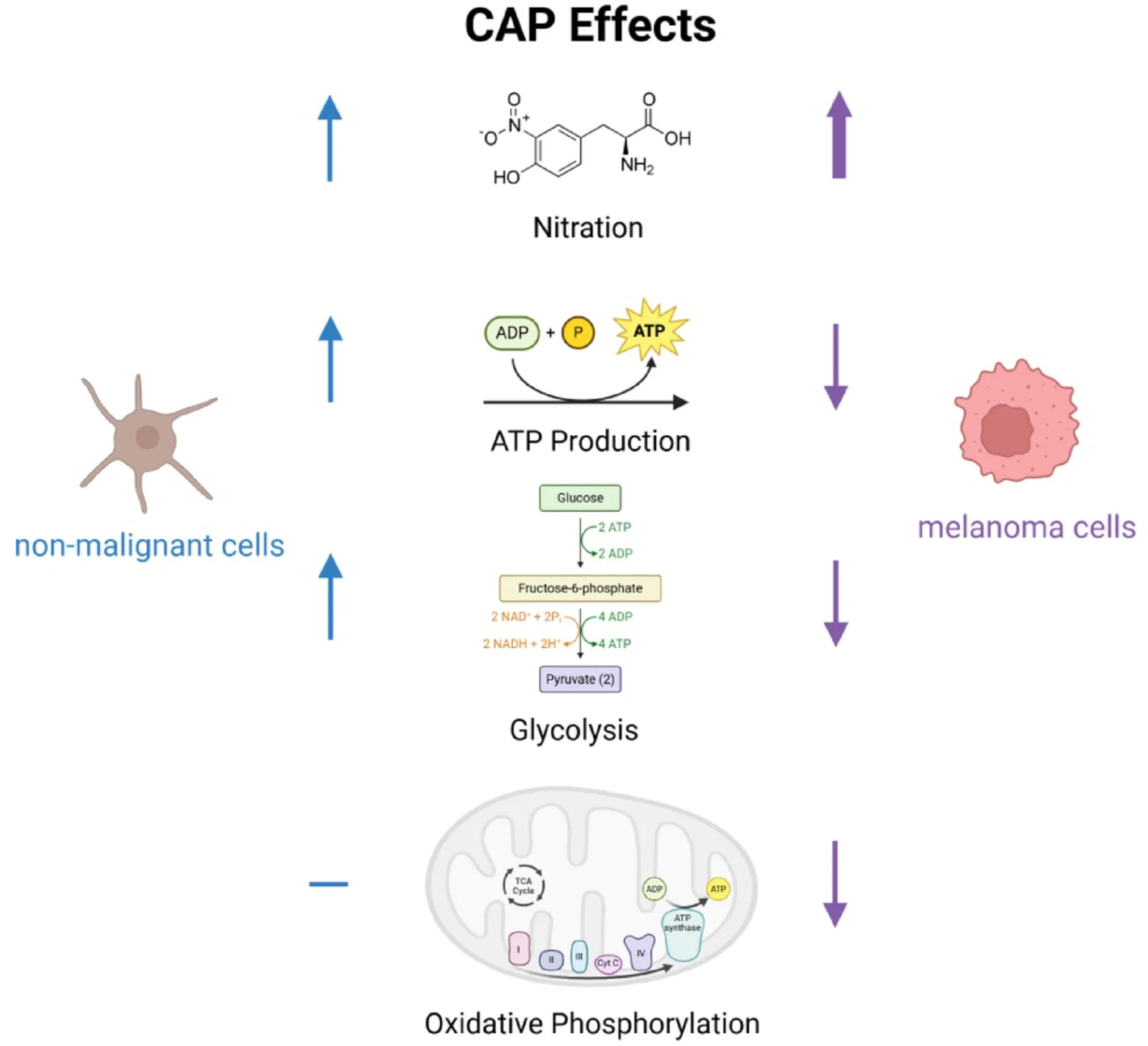
Graphical abstract about the CAP effects on the metabolism in melanoma vs. non-malignant cells. Created with Biorender.

### Statistical analysis

All assays were performed at least three independent times. The treatment was normalized to the respective control and statistically analyzed using an unpaired t-test in GraphPad Prism 10 (Version 10.4.1) from Graphpad Software Inc. (San Diego, USA). All bar charts illustrate the mean ± standard deviation. Furthermore, the RNA sequencing was analyzed using R (v 4.4.2) [28].

## Results

### CAP treatment inhibits metabolic processes in melanoma cells in a dose-dependent manner

To investigate oxidative phosphorylation and glycolysis, real-time measurement of the oxygen consumption rate (OCR) and extracellular acidification rate (ECAR) [36] of melanoma cell lines (Mel Im, Mel Juso) treated for 30 s or one minute with CAP was conducted. The “Mito Stress” test measurements of the CAP-treated melanoma cells showed a significant downregulation of the OCR and ECAR after CAP treatment. These effects even increased after a longer CAP treatment duration (Fig. 1A-D), indicating a dose-dependent suppression of the oxidative phosphorylation and glycolysis after CAP treatment in Mel Im and Mel Juso cells. Further analyses demonstrated a significant and again dose-dependent reduction of the basal, maximal, non-mito respiration, proton leak and spare respiratory capacity in CAP-treated Mel Im and Mel Juso (Fig. 1E-I), supporting the results in Fig. 1A-D. Next, we were interested in how CAP influences ATP production. Hence, we utilized an “ATP Rate” assay enabling to differentiate between ATP production from oxidative phosphorylation (mitoATP) and glycolysis (glycoATP) [37]. The results clearly revealed a significant downregulation of the ATP production rates in CAP-treated Mel Im and Mel Juso cells (Fig. 1J-K). This could be confirmed by a decreased ATP Rate Index, which is described as the ratio of the mitoATP production rate to the glycoATP production rate (Fig. 1L). These findings demonstrated that oxidative phosphorylation is slightly more affected by CAP than glycolysis in melanoma cells.

### CAP treatment induces gene and metabolite expression changes in melanoma cells

The following assays were conducted with one minute CAP-treated cells, because of the higher suppression of the metabolism compared to 30 s treated cells (Fig. 1). Longer durations of CAP treatment were not analyzed in these result in induction of apoptosis [25]. The subsequent objective was to investigate which metabolites are differentially regulated by CAP (metabolomics data see supplement 3). To achieve this, we performed an untargeted metabolomics analysis one hour after CAP treatment and control treatment in Mel Juso. The levels of several metabolites decreased after CAP, including lactate, fumarate, glutamic acid, nicotinamide adenine dinucleotide (NAD), biotin, proline, and carnitine (Fig. 2A). This suggests that CAP influences numerous important metabolic processes, including lipid metabolism, amino acid metabolism, glycolysis, oxidative phosphorylation, as well as cofactors that are crucial for various metabolic processes. Notably, guanosine was the only metabolite that showed a significant concentration increase in response to CAP (Fig. 2A). If we consider tendentially enhanced molecules like adenosine and hypoxanthine (see supplement 3), which are intermediate products in the breakdown of purine nucleotides to uric acid [38], it seems like the cells degraded ATP and GTP nucleotides since there was not enough energy to regenerate ATP and GTP due to the impaired metabolism. Additionally, an analysis using MetaboAnalyst 6.0 demonstrated 25 enriched or rather dysregulated metabolic pathways. The most significantly regulated pathway was the malate-aspartate shuttle, which is important for the translocation of NADH into the mitochondrial matrix for the oxidative phosphorylation [39], followed by the Warburg effect and citric acid cycle. In agreement, the amino acid and lipid metabolisms were deregulated after CAP treatment (Fig. 2B). These findings support the hypothesis of a suppressed metabolic state in melanoma cells after CAP treatment.

To unravel the underlying molecular mechanism of this CAP-induced suppression of the overall energy level and metabolism in melanoma, we conducted an RNA sequencing analysis. Therefore, we utilized CAP and control-treated Mel Im and Mel Juso cells after one hour and 24 h post-treatment incubation, respectively, to elucidate early and late-response effects of CAP. The volcano plots illustrate the resulting differentially expressed genes, in which the most significant ones (padj < 0.1; log2FC > 1.5) are labeled. Interestingly, the magnitude of differential gene expression (log2FC) is overall reduced after 24 h compared to one hour treated melanoma cells (Fig. 3A-D). Probably, because the early time point captures diverse acute transcriptional changes by CAP. On the contrary, at 24 h, the remaining cells may have adapted to the CAP-induced transcriptional changes (see tables of differentially expressed genes in supplement 5).

Moreover, one hour after CAP treatment, the genes PPP1R10, HSPA6 and HSPA1B were upregulated and CYR61 and TRIB1 were downregulated, while after 24 h only SLC7A11 was increased in both cell lines (Fig. 3A-D). This suggests that the anti-cancer CAP effect (see supplement 1b), especially on the metabolic level, was happening immediately after treatment, which was also supported by the metabolomic data of CAP-treated Mel Juso cells after one hour post-treatment incubation (Fig. 2).

Additionally, the heat shock protein family A member 6 (HSPA6), which is often induced under stress and seems to be associated with lipid and amino acid metabolism [40], showed a significant upregulation, which was also depicted in the heat map one hour after CAP treatment (see supplement 1d-e). This high gene expression of HSPA6 could also be observed after 24 h post-treatment in Mel Im cells (Fig. 3B). However, Mel Juso cells showed the same trend with regard to HSPA6 gene expression, but not as pronounced as in Mel Im cells. (see normalized counts, supplement 4).

To investigate this differential gene expression in more detail, a gene set enrichment analysis (GSEA) for the identification of associated biological processes was performed. Figure 3E and F illustrate regulated hallmark gene sets. Notably, metabolic processes like xenobiotic metabolism and bile acid metabolism were upregulated in both Mel Im and Mel Juso one hour after CAP treatment. Furthermore, genes regulating fatty acid metabolism were enriched in Mel Juso one hour after CAP (Fig. 3E). The early gene response (one hour) after CAP treatment appeared to be a compensation to regulate the metabolic state of the Mel Im and Mel Juso cells, considering that the cells were stressed by CAP. The long-term CAP effect (24 h) was affiliated with an enriched apoptosis rate and inflammatory response. After 24 h many metabolic pathways were downregulated like oxidative phosphorylation, fatty acid metabolism, cholesterol homeostasis in both cell lines and glycolysis in Mel Juso (Fig. 3F). This observation is in accordance with the previous results (Figs. 1 and 2).

### CAP treatment disrupts the uptake of important metabolites in melanoma cells

Furthermore, to validate the RNA sequencing findings the expression of selected genes was analyzed via qPCR on newly generated samples (Fig. 4). Since a downregulation of myc targets was observed in the GSEA analyses in both cell lines (Fig. 3E-F), we hypothesized that the activity or expression of c-myc and ESR1, which are regulators of the metabolism by inducing expression of metabolism-promoting genes [41, 42], may be downregulated. Thus, the mRNA expression of ESR1, c-myc and target genes (glucose transporter protein-type 1 (GLUT1), -type 4 (GLUT4) and hexokinase 2 (HK2)) were investigated via qPCR. If CAP dampened their expression, a metabolic slowdown would result. However, the data presented a contrasting narrative. Across both lines, CAP did not reduce mRNA levels of ESR1, c-myc, or their targets GLUT1, GLUT4, and HK2 (Fig. 4A-B, D-F). Some myc target genes were even significantly upregulated at some time points after CAP in one or both cell lines. GLUT1 mRNA expression was elevated in Mel Im at all time points after CAP, while the expression of GLUT4 was first significantly elevated and then downgraded after a longer incubation in Mel Juso and lastly HK2 mRNA expression was either upregulated or not regulated by CAP (Fig. 4D-F). Thus, CAP does not appear to suppress metabolism by shutting down these genes at the transcriptional level. Instead, CAP-treated Mel Im and Mel Juso cells may even mount a compensatory response, enhancing metabolism-promoting genes to counter CAP-induced metabolic stress.

One of the strongest regulated genes identified by differential expression analysis of the RNA sequencing data, which is known to be induced by stress and was mentioned earlier, (Fig. 3A-C) was HSPA6. The gene expression change of HSPA6 was validated via qPCR and showed an up to 15-fold upregulation after CAP treatment (Fig. 4C). This elevation could not be confirmed on protein level via western blot analysis, since the de novo protein synthesis was significantly impaired early after CAP treatment in Mel Im and Mel Juso (Fig. 4N-O). Overall, it appeared that the illustrated results of the RNA sequencing analysis are more likely a reaction to the CAP effects rather than the primary cause for the downregulation of metabolic processes.

To further investigate possible molecular drivers responsible for the observed metabolic changes after CAP-treatment in melanoma we focused on the mRNA expression of levels of sodium dependent multivitamin transporter (SMVT), a transporter for biotin and pantothenate [43], monocarboxylate transporter 1 (MCT-1) and 4 (MCT-4), which are transporters for monocarboxylates like lactate [44]. Biotin, pantothenate and lactate were decreased after CAP treatment in the metabolite analysis (Fig. 2A). Notably, the gene expression of these transporters was not significantly deregulated after CAP in both cell lines (Fig. 4G-I). Therefore, the downregulation of the essential cofactors (Fig. 2A) cannot be explained by differential gene expression of the respective transporters.

To examine why various metabolic pathways were affected after CAP treatment (Fig. 2A-B), an uptake assay of glucose, glutamine and glutamate was performed for CAP-treated melanoma cells after four, seven hour and 24 h incubation, to view the timeline of the uptake, by measuring the metabolite concentrations in the supernatants. The concentration of glucose was significantly higher at seven and 24 h after CAP for both melanoma cell lines (Fig. 4L), while the glutamine and glutamate concentration were significantly higher only after 24 h (Fig. 4J-K). Since the mRNA expression of GLUT1 and GLUT4 was not downregulated in both Mel Im and Mel Juso (Fig. 4D-E), the impaired uptake of important metabolites is most likely due to posttranslational modifications and dysfunctionality of already synthesized proteins e.g. by protein oxidation via RONS resulting in protein carbonylation. The protein carbonylation content was measured one hour after CAP treatment in Mel Im and Mel Juso. Unexpectedly, the carbonyl content did not differ between control and CAP-treated cells (Fig. 4M). Therefore, another post-translational modification was taken into consideration. In the past, our research group already demonstrated that nitrotyrosine modification of proteins happens after CAP treatment in Mel Im and Mel Juso [45]. Thus, we predict that protein nitration led to an impaired metabolism by altering a variety of proteins including transporters for metabolites.

### CAP-treatment induces glycolysis in non-malignant cells and nitration in melanoma and non-malignant cells

Real-time metabolic measurements were conducted with non-malignant cells (NHEM, Bj1) to compare the effects of CAP on their metabolism. Since CAP is known to have rather positive effects on non-malignant cells [6], it was expected that melanocytes and fibroblasts would either remain unchanged or experience an increase in their metabolism after treatment. The OCR (oxygen consumption rate) was not significantly altered by CAP (Fig. 5A-B). Interestingly, only the ECAR (extracellular acidification rate) showed a significant increase (Fig. 5C-D), indicating a higher glycolytic rate in NHEM and Bj1 cells after CAP treatment. This finding is further supported by the ATP Rate assay, which revealed an increase in total ATP production (Fig. 5E).

However, the question of why the CAP-induced metabolic effects differ between malignant and non-malignant cells needs to be answered. qPCR analysis of CAP-treated NHEM depicts that the enhanced glycolysis via CAP is not based on elevated glycolytic gene expression, since the mRNA expression of GLUT4 (Fig. 5F), GLUT1 (Fig. 5G) and HK2 (Fig. 5H) were not significantly increased in both NHEM and Bj1 cells. Therefore, it is most likely that the activity of important glycolytic proteins is increased.

Interestingly, the mRNA expression of the heat shock protein HSPA6 was increased up to 70-fold in CAP-treated NHEM and Bj1 cells compared to the control (Fig. 5I). Overall, the HSPA6 mRNA expression is even higher in NHEM and Bj1 cells versus Mel Im and Mel Juso cells after CAP treatment (Fig. 4C). It seems like the protective reaction against RONS is stronger induced in NHEM and Bj1 cells and is working properly since we could not detect a negative influence of CAP in these cells. Furthermore, the proteins of NHEM and Bj1 cells were also nitrated after one hour CAP treatment as in the Mel Im and Mel Juso cells. Still, a difference between malignant and non-malignant cells was detected. The NHEM and Bj1 cells demonstrated a lower increase in nitrotyrosine after CAP treatment via western blot analysis than the Mel Im and Mel Juso cells (Fig. 5J-K). Ponceau staining of these western blots can be viewed in supplement 1c. In summary, fewer proteins were affected by CAP-induced RONS and the NHEM and Bj1 cells appeared to be better prepared against such stressors. The cell reaction triggered by CAP even led to an increase in glycolysis, therefore CAP had a positive effect on non-malignant cells (NHEM, Bj1).

## Discussion

In this study we aimed to investigate the effects of CAP treatment on metabolic processes in melanoma cells, since only few studies focusing on CAP effects in context of cell metabolism exist so far. Our results clearly showed a decrease in glycolysis, oxidative phosphorylation and ATP production in melanoma cells after CAP treatment, demonstrating CAP induced metabolic changes in vitro. This finding is supported by the observed reduction of important metabolites from various metabolic pathways and the suppressed uptake of glucose, glutamine and glutamate. Other research groups have suggested that the metabolism is impaired in CAP-treated cancer cells. One group discovered a decline in the glutaminase activity causing a toxic accumulation of glutamine in leukemia cells after CAP [46]. Also, miRNA regulation by ROS after CAP could affect the metabolism [47]. Additionally, Murakami et al. revealed that CAP led to changes in the metabolic activity and state of cells [48]. In our study NAD was significantly decreased one hour after CAP treatment in melanoma cells. This appears to be a general CAP effect on cancer cells, as another group also discovered a decreased NAD(P)H concentration via fluorescence microscopy of breast cancer cells [49]. Moreover, the, in this study, proven suppression of the metabolic state is probably one additional reason for the CAP­induced apoptotic effect on malignant cells.

In contrast to melanoma cells, we could clearly reveal that CAP treatment does not negatively influence the metabolism of fibroblasts and melanocytes. Miao Tian et al. discovered a correlation between CAP-treated cancer cells and the purine degradation pathways. The amount of the metabolites xanthine, inosine and hypoxanthine, which are intermediates involved in the purine degradation [50] were significantly increased in the CAP-treated melanoma cell line A375 compared to keratinocytes [51]. Our group has also observed a tendency towards increase in xanthine and hypoxanthine concentration in Mel Juso. Interestingly, the xanthine-oxidoreductase, which converts hypoxanthine to xanthine, is often expressed in skin cancer and could lead to a higher RONS production [52].

The results of this study indicate CAP-induced inhibition of metabolic processes probably based on posttranslational protein modifications like nitration at tyrosine residues. It is known that CAP led to protein nitration [25], but now we could show for the first time a significant increase in protein nitration after CAP treatment of melanoma cells compared to melanocytes and fibroblasts. RONS induce post-translational modifications. Not only nitration, as investigated in this study, but also for example the oxidation of cysteine and methionine residues and carbonylation can take place in CAP-treated cells. Such modifications can alter the activity, stability, localization and interaction between proteins [53], influencing redox-sensitive pathways like the glycolysis and oxidative phosphorylation, which results in a decreased ATP production in cancer cells, as indicated in this study. Since many tumor cells have a higher physiological basal RONS concentrations, compared to non-malignant cells [54], the “threshold” for RONS-mediated DNA damage and cell death is reached earlier in cancer cells compared to non-malignant cells [55]. Therefore, non-malignant cells are more likely to restore redox balance after CAP. This difference is one reason for the anti-tumor specific effect of CAP, which we also demonstrated in this study.

Nitration is probably the most common modification induced by CAP, since large amounts of nitrite are generated during CAP treatment and significantly contribute to its effect [25]. The nitrogen required for this reaches the cell via CAP-induced RONS. Furthermore, extracellular acidification by CAP promotes the nitration even further [56]. Normally, glutathione inhibits the formation of nitrotyrosine [57], however glutathione was significantly decreased in CAP-treated Mel Juso. For example, Logan et al. revealed that nitration at different tyrosine sites in HSP90 led to different metabolic effects. Nitration at one site decreased the oxidative phosphorylation and at the second enhanced the glycolysis [58]. HSP90 is like HSPA6, which is significantly upregulated in our study after CAP treatment and in others [59, 60], a chaperone [61] to protect a cell against damage [62]. Heat shock proteins are often targets for nitration [63, 64]. Furthermore, mitochondrial proteins are sensitive for protein nitration [64]. Another study even mentioned that high concentrations of nitrotyrosine induce mitochondrial damage, while low concentration increased the oxygen consumption [65].

The strength of the reaction to CAP-derived RONS not only depends on their amount, but also on their capability to enter the cells. Aquaporins are known to facilitate the transport of RONS [5]. Cancer cells express more aquaporins, which lead to a higher uptake of RONS [66]. However, there are also further factors that can influence the CAP effect: The lipid composition of the membrane [4] and a transient CAP-induced membrane permeabilization, often termed as plasmaporation, can lead to differential RONS uptake [67]. Malignant and non-malignant cells often differ in lipid composition, e.g. cancer cells have less cholesterol. Therefore, they are more susceptible to lipid peroxidation and CAP-derived RONS [68]. Such differences can contribute to cell type-specific RONS accumulation and thus to selective CAP effects. While this mechanism was not examined in the present study, it may add to the observed cellular responses following CAP treatment.

Therefore, the discovered differential quantity of nitration, demonstrated in this study, seemed to lead to the CAP-induced suppression of the metabolism in cancer cells only. Furthermore, our findings indicate that CAP would be a good tool for combination therapy against cancer. One study has shown that a combination of CAP with metformin, a mitochondrial complex I inhibitor, results in synergistic cytotoxicity, since metformin sensitized glioma cells to CAP-induced oxidative stress [69]. Moreover, the combination of CAP with temozolomide or doxorubicin can increase the metabolic stress level for cancer cells [70, 71]. The idea of anti-cancer agent combination with CAP is promising, but since CAP is limited with its penetration depth, it is more forward looking to use CAP in the operation room after a tumor removal [19].

## Conclusion

Taken together, we discovered a dose-dependent downregulation of different metabolic pathways including glycolysis and oxidative phosphorylation in CAP-treated melanoma cells, while the glycolysis and ATP production were elevated after the treatment in non-malignant cells. All cell lines demonstrated an increased mRNA expression level of the heat shock protein HSPA6 and increased levels of nitrotyrosine already one hour after CAP treatment. Interestingly, malignant cells demonstrated a higher protein nitration rate. As such molecular changes affect the functionality of several proteins, it is likely that the differential protein nitration found in melanoma and non-malignant cells contributes to the cancer-specific impairment of metabolic processes, along with other CAP effects. Our findings are summarized in Fig. 6.

## Supplementary Information

Supplementary Material 1.

Supplementary Material 2.

Supplementary Material 3.

Supplementary Material 4.

Supplementary Material 5.

## Data Availability

The datasets used and/or analyzed during the study are available from the corresponding author on request.

## Abbreviations

CAP: Cold atmospheric plasma
ECAR: Extracellular acidification rate
FCCP: Carbonylcyanide-4-(trifluoromethoxy)phenylhydrazone
GAPDH: Glycerinaldehyde-3-phosphate dehydrogenase
GSEA: Gene set enrichment analysis
HPG: L-Homopropargylglycine
HRP: Horseradish peroxidase
log2FC: Log2FoldChange
MCT-1: Monocarboxylate transporter 1
MCT-4: Monocarboxylate transporter 4
MSigDB: Molecular signature database
NAD: Nicotinamide adenine dinucleotide
NFDM: Non-fat dried milk
NHEM: Normal human epidermal melanocytes
OCR: Oxygen consumption rate
padj: Adjusted p-value
RONS: Reactive oxygen and nitrogen species
Rot/AA: Rotenone/Antimycin A
SMVT: Sodium multivitamin transporter
