# Supplementary material for: Cold atmospheric plasma affects metabolic processes in malignant melanoma
Source: Cancer Cell Int. 2026 Aug 22;26:293. doi: 10.1186/s12935-026-04448-3 (PMC13499309; doi:10.1186/s12935-026-04448-3)
Supplement: Supplementary file 1 [file 12935_2026_4448_MOESM3_ESM.docx]

**Supplement 1**

*1a: Scheme of the experimental setup*

Abbreviations: d = treatment distance; t = treatment duration

*1b: Cell viability measurement for one minute CAP-treated cells*

*CAP treatment was normalized to the respective control and statistically analyzed using an unpaired t‑test; * p < 0.05; ** p < 0.005; *** p < 0.0005; n=3; mean ± standard deviation*

*1c: Western Blots 1h after CAP*

Order for every blot:

Bj1 Ctrl, Bj1 CAP, NHEM Ctrl, NHEM CAP, Mel Im Ctrl, Mel Im CAP, Mel Juso Ctrl, Mel Juso CAP

Nitrotyrosine	GAPDH	Ponceau

 n1

n2

n3

*1d: Heatmap of the top 10 % differential regulated genes in Mel Im and Mel Juso 1h after CAP treatment (analyzed via RNA sequencing)*

Red = upregulation

Blue = downregulation

*1e: Heatmap of the top 5 % differential regulated genes in Mel Im and Mel Juso 24h after CAP treatment (analyzed via RNA sequencing)*

Red = upregulation

Blue = downregulation
